# Algorithm and Distributed Computing for the Internet of Things

**DOI:** 10.3390/s20164513

**Published:** 2020-08-12

**Authors:** Juan A. Gómez-Pulido, Jorge Sá Silva, Takahiro Hara

**Affiliations:** 1 Department of Technologies of Computers and Communications, University of Extremadura, 10003 Caceres, Spain; 2 Department of Electrical and Computer Engineering, University of Coimbra, 3030-290 Coimbra, Portugal; sasilva@deec.uc.pt; 3 Department of Multimedia Engineering, Osaka University, Osaka 565-0871, Japan; hara@ist.osaka-u.ac.jp

## Abstract

The ongoing generalization of Internet of Things and its presence and application in multiple fields is generating a large amount of data that can be used to extract knowledge, among other purposes. In this context, algorithmic techniques and efficient computer systems provide an opportunity to successfully address efficient data processing and intelligent data analysis. As a result, multiple services can be improved, resources can be optimized and real-world problems of interest can be solved. This Special Issue on Algorithm and Distributed Computing for the Internet of Things gives the opportunity to know recent advances in the application of modern technologies hardware and software to the Internet of Things.

## 1. Introduction

There is a growing interest in applying technologies, such as the Internet of Things (IoT), derived from the intense deployment of Wireless Sensor Networks (WSNs) over recent decades, in relevant fields such as Industry 4.0, smart cities, and connected infrastructures. The use of IoT in these areas can lead to significant benefits, but it also generates a huge amount of data, which should be processed by a system to, in many cases, detect patterns or optimize certain processes of interest. To this end, big data, machine learning, and deep learning approaches are usually considered, leading to more complex and powerful systems than those of previous WSN applications, which also means new challenges to address. Moreover, for critical areas such as connected infrastructures, dependability, sustainability, and security issues should be addressed to ensure specific application requirements.

In this Special Issue, we have collected original high-quality articles, clearly focused on theoretical and implementation solutions for IoT, including intelligent approaches (machine learning, big data, and deep learning), network levels (edge, fog, and cloud), embedded systems, sensing devices, nonfunctional requirements (dependability, security, and sustainability), deployment strategies, and management platforms. The papers submitted to this Special Issue fitted some of the following topics, which were listed in the call for papers: Smart manufacturing and Industry 4.0; connected vehicles; smart cities; cognitive computing and deep learning; big data processing; edge computing and network intelligence, bringing the computation closer to the data; dependability (real-time, reliability, availability, safety); sustainability (low-power operation, energy management, energy harvesting); security and privacy; distributed and embedded computing for networked systems; applications, deployment, and management in smart environments; human behavior and context-aware aspects; prediction models; and optimization and metaheuristics.

## 2. Summary of the Contributions in This Special Issue

Although each paper published in this Special Issue covers different topics, we can identify four groups where the papers can be classified according to their main focus: Privacy and security; efficiency and performance; algorithms and intelligence; and protocols, networks and infrastructures. However, some of these papers could be classified into more than one of these groups.

With regard to privacy and security, we find two contributions.

The first article in this group is entitled “Location Privacy Protection in Distributed IoT Environments Based on Dynamic Sensor Node Clustering” [1], by Konstantinos Dimitriou and Ioanna Roussaki. This research focuses on the protection of privacy in IoT environments. Without guaranteing the privacy of sensitive data collected and shared over IoT infrastructures, many applications can be discarded by the users. This article introduces a dynamic clustering algorithm that aims to protect the privacy of location information across Data Centric Sensor Networks (DCSNs) that monitor the location of mobile objects in IoT systems. This algorithmic proposal significantly outperforms existing solutions focusing on enhancing the privacy of location information in IoT.

The second article in this group is entitled “Pseudo-Random Encryption for Security Data Transmission in Wireless Sensor Networks” [2], by Liang Liu, Wen Chen, Tao Li and Yuling Liu. This work focuses on the security of wireless sensor networks (WSN), through a lightweight security scheme to protect the confidentiality of data transmission between sensors and an ally fusion center (AFC) over insecure links. This proposal solves the typical security problem of WSN’s binary hypothesis testing of a target’s state, where sensors are divided into flipping and non-flipping groups according to the outputs of a pseudo-random function which was held by sensors and the AFC. Then in order to prevent an enemy fusion center (EFC) from eavesdropping, the binary outputs from the flipping group were intentionally flipped to hinder the EFC’s data fusion. Accordingly, the AFC performed inverse flipping to recover the flipped data before data fusion. This proposed scheme was extended to a more common scenario with multiple scales of sensor quantification and candidate states.

With regard to efficiency and performance, we find other two contributions.

The first article in this group is entitled “Mapping Neural Networks to FPGA-Based IoT Devices for Ultra-Low Latency Processing” [3], by Maciej Wielgosz and Michał Karwatowski. This research exploits the power of reconfigurable hardware to implement algorithms that run efficiently thanks to the intrinsic parallelism provided by field programmable gate arrays (FPGA) devices. Particularly, this computing resource can solve situations in IoT infrastructures where the response time in the access to knowledge is more critical to ensure Quality of Service than the quality of the answer. To this end, the authors propose a methodology to map the models to FPGA in order to reduce the latency. Multi-objective covariance matrix adaptation evolution strategy (MO-CMA-ES) was employed along with custom scores for sparsity, bit-width of the representation and quality of the model. Furthermore, the authors created a framework which enables mapping of neural models to FPGAs.

The second article in this group is entitled “Distributed Egocentric Betweenness Measure as a Vehicle Selection Mechanism in VANETs: A Performance Evaluation Study” [4], by Ademar T. Akabane, Roger Immich, Richard W. Pazzi, Edmundo R. M. Madeira and Leandro A. Villas. This work studies the performance of the vehicle selection mechanism (VSM) that is applied to find, in a distributed fashion, the best-ranked vehicles in the network after each topology change. To this end, the authors consider the egocentric concept, which analyses the social environment surrounding individuals by using locally available knowledge of the topology. The research analyzes if egocentric measures can be a viable alternative for implementing a VSM. According to the performance results, VSM seems to be very useful for vehicular ad-hoc network (VANET) applications.

With regard to algorithms and intelligence, we find a wide area of interest where this Special Issue provides seven contributions.

The first article in this group is entitled “DNN-MVL: DNN-Multi-View-Learning-Based Recover Block Missing Data in a Dam Safety Monitoring System” [5], by Yingchi Mao, Jianhua Zhang, Hai Qi and Longbao Wang. This work deals with missing data from sensors, specially when they are largely deployed and are constrained by batter power, memory, and computational capacity, among other factors. Particularly, the problem arises when some sensors lose readings at consecutive time slots. In this case, the accuracy of real-time monitoring and the performance on the data analysis in the WSNs are affected, specially when existing solutions to the missing data filling cannot well uncover the complex non-linear spatial and temporal relations. In this work, the authors propose a DNN (Deep Neural Network) multi-view learning method (DNN-MVL) to fill the successive missing readings. This technique has been validated with success from experiments on large-scale real-world datasets, outperforming all of the baseline methods.

The second article in this group is entitled “Coverage-Balancing User Selection in Mobile Crowd Sensing with Budget Constraint” [6], by Yanan Wang, Guodong Sun and Xingjian Ding. Mobile crowd sensing (MCS) is a powerful technique to achieve urban-scale sensing and data collection for IoT. In the MCS campaign, the smart mobilephone users can detect their surrounding environments with their on-phone sensors and return the sensing data to the MCS organizer. This research focuses on the coverage-balancing user selection (CBUS) problem with a budget constraint. The authors first propose a novel coverage balance-based sensing utility model for solving the CBUS problem. Next, they define the CBUS problem under the proposed sensing utility model. Because of the NP-hardness of the CBUS problem, a heuristic-based algorithm (MIA) is applied to determine a preliminary subset of users from all the available users and then adjusts this user subset to improve the budget implementation. This proposal show good results both in coverage balance and in the total coverage area.

The third article in this group is entitled “Optimizing Movement for Maximizing Lifetime of Mobile Sensors for Covering Targets on a Line” [7], by Peihuang Huang, Wenxing Zhu and Longkun Guo. The research starts from a set of sensors distributed on the plane and a set of Point of Interests (POIs) on a line segment. The goal of a a WNS is to cover the POIs by the sensors, such that each POI is monitored by at least one sensor. For balancing the energy consumption, the authors study the min-max line barrier target coverage (LBTC) problem which aims to minimize the maximum movement of the sensors from their original positions to their final positions at which the coverage is composed. As the radius of the sensors are non-uniform integers, LBTC problem is NP-hard, even considering the sensors distributed on the line segment instead of the plane. The problem is then solved by an exact algorithm that runs produces an optimal solution in reasonable time.

The fourth article in this group is entitled “Wireless Charging Deployment in Sensor Networks” [8], by Wei-Yu Lai and Tien-Ruey Hsiang. This work focuses on the charging schemes used in mobile wireless chargers to prolong the lifespan of a WSN. Altough other works focus on charging each sensor from a single position, then optimizing the moving paths of the chargers, the authors work close to real-world when considering that a wireless charger may charge the same sensor from several positions in its path. Then, the research effort is addressed to minimize both the number of charging locations and the total required charging time. To this end, two charging plans are developed. The first plan considers the charging time required by each sensor and greedily selects the charging service positions. The second one minimizes the number of charging positions and assigns minimum charging times to every position according to the charging requirements of the nearby sensors.

The fifth article in this group is entitled “A oneM2M-Based Query Engine for Internet of Things (IoT) Data Streams” [9], by Putu Wiramaswara Widya, Yoga Yustiawan and Joonho Kwon. This paper presents a query engine, called OMQ, based on the standard oneM2M (one machine-to-machine) for the interoperability of the architecture and protocols of IoT. This engine was programmed by using JavaScript Object Notation (JSON) to provide a real-time processing over IoT data streams. The authors also propose efficient query processing algorithms, which utilizes the oneM2M architecture consisting of the IoT node and the infrastructure node. Since the query processing algorithms are implemented as the hybrid infrastructure-edge processing, user queries can be executed efficiently in each IoT node rather than only in the infrastructure node. As result, OMQ reduces the query processing time and the network bandwidth.

The sixth article in this group is entitled “Natural Computing Applied to the Underground System: A Synergistic Approach for Smart Cities” [10], by Clemencio Morales Lucas, Luis Fernando De Mingo López and Nuria Gómez Blas. The authors apply Swarm Intelligence to model the intelligence of smart cities, by considering a swarm of digital telecommunication networks (the nerves), ubiquitously embedded intelligence, sensors and tags, and software. The goal is to obtain a complete control of the Urban Public Transport Systems (UPTS). To this end, the paper presents an algorithmic approach based on Ant Colony Optimization and Fireworks algorithms.

The last article in this group is entitled “Connected Vehicle as a Mobile Sensor for Real Time Queue Length at Signalized Intersections” [11], by Kai Gao, Farong Han, Pingping Dong, Naixue Xiong and Ronghua Du. This research solves the problem of existing models’ complexity and information redundancy in intelligent transportation system (ITS) and vehicle to X (V2X), where the connected vehicle collects useful traffic information, such as queue length at intersections. To this end, the authors proposes a queue length sensing model based on V2X technology, which consists of two sub-models based on shockwave sensing and back propagation neural network sensing. The model obtains state information of the connected vehicles, analyzes the queue, and calculates the velocity of the shockwave to predict the queue length of the subsequent unconnected vehicles. After that, the neural network is trained with historical connected vehicle data, and a sub-model based on the neural network predicts the real-time queue length. Finally, the queue length at the intersection is determined by combining the sub-models by variable weight. As result, the sensing accuracy of the combined model is proportional to the penetration rate of connected vehicles, and sensing of queue length can be achieved even in low penetration rate environments.

The last group focuses mainly on protocols, networks and infrastructures. We find two contributions in this group.

The first article in this group is entitled “An Optimized Probabilistic Delay Tolerant Network (DTN) Routing Protocol Based on Scheduling Mechanism for Internet of Things (IoT)” [5], by Yuxin Mao, Chenqian Zhou, Yun Ling and Jaime Lloret. This work focuses on delay tolerant networks (DTN), where the efficient data transmission is an important issue for IoT applications. Particullarly, it important to improve delivery rate and optimize delivery delay with low overhead. To this end, the authors propose a new routing protocol, called Scheduling-Probabilistic Routing Protocol using History of Encounters and Transitivity (PROPHET), which allows calculating the delivery predictability according to the encountering frequency among nodes. This protocol is evaluated by using two scheduling mechanisms, and is compared with other routing protocols in an Opportunistic Network Environment (ONE) simulator. Thus, the proposed scheduling protocol achieves better performances in key aspects compared with the existing protocols.

The second article in this group is entitled “Design and Analysis of a General Relay-Node Selection Mechanism on Intersection in Vehicular Networks” [12], by Dun Cao, Bin Zheng, Jin Wang, Baofeng Ji and Chunhai Feng. Vehicular networks (VNET) can extend the coverage of a message by means of relay nodes, which can be selected according to the prior information regarding the road structure. However, the non-line-of-sight (NLOS) communication in the intersection scenarios and diverse shapes for the intersection hamper the design of a general relay-node selection on intersection. This problem is solved by the authors after building a model to describe the general intersection, and proposing an algorithm for selecting relay-nodes. The performance of this selection algorithm on the intersection are explored in terms of message dissemination speed and Packet Delivery Ratio (PDR).

Finally, we thank all authors who have submitted their manuscripts to this Special Issue for considering Sensors and the reviewers for their hard work with the reviewing process.
